# Effective Accentuation of Voltage-Gated Sodium Current Caused by Apocynin (4′-Hydroxy-3′-methoxyacetophenone), a Known NADPH-Oxidase Inhibitor

**DOI:** 10.3390/biomedicines9091146

**Published:** 2021-09-03

**Authors:** Tzu-Hsien Chuang, Hsin-Yen Cho, Sheng-Nan Wu

**Affiliations:** 1 Department of Physiology, National Cheng Kung University Medical College, No. 1, University Road, Tainan 70101, Taiwan; fytg55qq@gmail.com (T.-H.C.); lilyzhou861126@gmail.com (H.-Y.C.); 2 Institute of Basic Medical Sciences, National Cheng Kung University Medical College, Tainan 70101, Taiwan

**Keywords:** apocynin (4′-Hydroxy-3′-methoxyacetophenone), NADPH-dependent oxidase (NOX), voltage-gated Na^+^ current, persistent Na^+^ current, erg-mediated K^+^ current, current kinetics, voltage-dependent hysteresis, electrically excitable cell

## Abstract

Apocynin (*aPO*, 4′-Hydroxy-3′-methoxyacetophenone) is a cell-permeable, anti-inflammatory phenolic compound that acts as an inhibitor of NADPH-dependent oxidase (NOX). However, the mechanisms through which *aPO* can interact directly with plasmalemmal ionic channels to perturb the amplitude or gating of ionic currents in excitable cells remain incompletely understood. Herein, we aimed to investigate any modifications of *aPO* on ionic currents in pituitary GH_3_ cells or murine HL-1 cardiomyocytes. In whole-cell current recordings, GH_3_-cell exposure to *aPO* effectively stimulated the peak and late components of voltage-gated Na+ current (I_Na_) with different potencies. The EC_50_ value of *aPO* required for its differential increase in peak or late I_Na_ in GH_3_ cells was estimated to be 13.2 or 2.8 μM, respectively, whereas the K_D_ value required for its retardation in the slow component of current inactivation was 3.4 μM. The current–voltage relation of I_Na_ was shifted slightly to more negative potential during cell exposure to *aPO* (10 μM); however, the steady-state inactivation curve of the current was shifted in a rightward direction in its presence. Recovery of peak I_Na_ inactivation was increased in the presence of 10 μM *aPO*. In continued presence of *aPO*, further application of rufinamide or ranolazine attenuated *aPO*-stimulated I_Na_. In methylglyoxal- or superoxide dismutase-treated cells, the stimulatory effect of *aPO* on peak I_Na_ remained effective. By using upright isosceles-triangular ramp pulse of varying duration, the amplitude of persistent I_Na_ measured at low or high threshold was enhanced by the *aPO* presence, along with increased hysteretic strength appearing at low or high threshold. The addition of *aPO* (10 μM) mildly inhibited the amplitude of erg-mediated K+ current. Likewise, in HL-1 murine cardiomyocytes, the *aPO* presence increased the peak amplitude of I_Na_ as well as decreased the inactivation or deactivation rate of the current, and further addition of ranolazine or esaxerenone attenuated *aPO*-accentuated I_Na_. Altogether, this study provides a distinctive yet unidentified finding that, despite its effectiveness in suppressing NOX activity, *aPO* may directly and concertedly perturb the amplitude, gating and voltage-dependent hysteresis of I_Na_ in electrically excitable cells. The interaction of *aPO* with ionic currents may, at least in part, contribute to the underlying mechanisms through which it affects neuroendocrine, endocrine or cardiac function.

## 1. Introduction

Apocynin (*aPO*, 4′-Hydroxy-3′-methoxyacetophenone), a polyphenolic compound, is a naturally occurring ortho-methoxy-substitued catechol isolated from a variety of plant sources, including *Apocynum cannabinum*, *Pierorhiza kurroa*, and so on [1]. Of note, this compound has been widely used as a selective inhibitor of NADPH-dependent oxidase (NOX) [2,3,4,5]. Alternatively, it has been recognized to be one of the most promising drugs in a variety of pathophysiological disorders, such as inflammatory and neurodegenerative diseases, glioma, and cardiac failure [1,3,5,6,7,8,9,10,11]

*aPO* has been recently shown to ameliorate cardiac function (e.g., structural remodeling) in heart failure [6,7,11,12,13]. Pituitary cells were previously demonstrated to be expressed in the activity of NOX [14,15]. *aPO* has been reported to blunt the progression of neuroendocrine alterations induced by social isolation, which were thought to be mainly through its inhibition of NOX activity [16]. However, whether *aPO* exercises any modifications on ionic currents remains largely unknown.

The voltage-gated Na^+^ (Na_V_) channels, nine subtypes of which are denoted Na_V_1.1 through Na_V_1.9, belong to the larger protein superfamily of voltage-dependent ion channels and their activity plays an essential role in the generation and propagation of action potentials (APs) in electrically excitable cells. The Na_V_ channels contain four homologous domains (DI-DIV), each of which consists of a six α-helical transmembrane domain (S1–S6) and a reentry P loop between S5 and S6. Na_V_1.5 channels primarily underlie AP initiation and propagation in the heart, these channels have also been shown to be critical determinants of AP duration, particularly in the setting of certain arrhythmias (e.g., LQT-3 syndrome) [17,18]. Previous studies have demonstrated the ability of *aPO* to attenuate angiotensin II-induced activation of epithelial Na^+^ channels in human umbilical vein endothelial cells as well to blunt activation of these channels caused by epidermal growth factor, insulin growth factor-1 or insulin [19,20]. However, the issue of how *aPO* or other related compounds could perturb the amplitude or kinetic gating of transmembrane ionic currents (e.g., voltage-gated Na^+^ current [I_Na_]) still remains unmet.

Therefore, in the present study, the electrophysiological effects of *aPO* and other related compounds in pituitary GH_3_ cells and in HL-1 atrial cardiomyocytes were investigated. We sought to (1) evaluate whether the *aPO* presence has any effect on the amplitude, gating and voltage-dependent hysteresis (Vhys) of I_Na_ residing in GH_3_ cells; (2) compare the effect of other related compounds on the peak amplitude of I_Na_; (3) study the effect of *aPO* on erg-mediated K^+^ current in GH_3_ cells; and (4) investigate the effect of *aPO* on I_Na_ in HL-1 cardiomyocytes. Findings from this study, for the first time, provide distinctive evidence to show that, in addition to its effectiveness in suppressing NOX activity, the differential stimulation by *aPO* of peak and late I_Na_ may be engaged in varying ionic mechanisms underlying its perturbations on the functional activities of electrically excitable cells (e.g., GH_3_ or HL-1 cells).

## 2. Materials and Methods

### 2.1. Chemicals, Drugs and Solutions Used in the Present Work

Apocynin (*aPO*, NSC 2146, NSC 209524, acetovanillone, acetoguaiacone, 4′-Hydroxy-3′-methoxyacetophenone, 1-(4-Hydroxy-3-methoxyphenyl)ethanone, C_9_H_10_O_3_, CAS number: 498-02-2, https://pubchem.ncbi.nlm.nih.gov/compound/Acetovanillone (accessed on 16 September 2004)), methylglyoxal (MeG, acetylformaldehyde, pyruvaldehyde, pyruvic aldehyde), norepinephrine, superoxide dismutase (SOD), tefluthrin (Tef), tetraethylammonium chloride (TEA), and tetrodotoxin (TTX) were acquired from Sigma-Aldrich (Merck, Taipei, Taiwan), rufinamide (RFM, 1-[(2,6-difluorophenyl]-1H-1,2,3-triazole-4-carboxamide), E-4031 and ranolazine (Ran) were from Tocris (Union Biomed, Taipei, Taiwan), and esaxerenone (ESAX) was from MedChemExpress (Gene-chain, Kaohsiung, Taiwan). Unless noted otherwise, culture media (e.g., F-12 medium), horse serum, fetal bovine or calf serum, L-glutamine, and trypsin/EDTA were purchased from HyCloneTM (Thermo Fisher Scientific, Tainan, Taiwan), while all other chemicals were of laboratory grade and taken from standard sources.

The HEPES-buffered normal Tyrode’s solution used in this work had an ionic composition, comprising (in mM): NaCl 136.5, KCl 5.4, CaCl_2_ 1.8, MgCl_2_ 0.53, glucose 5.5, and HEPES 5.5, and the pH was adjusted with NaOH to 7.4. For measurements of I_Na_ or I_Na(P)_, we kept GH_3_ or HL-1 cells immersed in Ca^2+^-free, Tyrode’s solution in attempts to avoid the contamination of Ca^2+^-activated K^+^ currents and voltage-gated currents. To record K^+^ currents, we filled up the recording pipette with a solution containing (in mM): K-aspartate 130, KCl 20, KH_2_PO_4_ 1, MgCl_2_ a, Na_2_ATP 3, Na_2_GTP 0.1, EGTA 0.1, HEPES 5, and the pH was titrated to 7.2 by adding KOH, while to measure I_Na_ or I_Na(P)_, we substituted K^+^ ions in internal pipette solution for equimolar Cs^+^ ions and the pH in the solution was adjusted to 7.2 by adding CsOH. All solutions used in this study were prepared using demineralized water from Milli-Q purification system (Merck). On the day of experiments, we filtered the bathing or filling solution and culture medium by using Acrodisc^®^ syringe filter with a 0.2-μm pore size (Bio-Check, Tainan, Taiwan).

### 2.2. Cell Preparations

These are provided in the Supplemental Materials mentioned in previous studies [21,22].

### 2.3. Electrophysiological Measurements

Shortly before experiments, we dispersed cells with 1% trypsin/EDTA solution and an aliquot of cell suspension was quickly placed in a custom-built chamber affixed to the stage of a CKX-41 inverted microscope (Olympus; Taiwan Instrument, Tainan, Taiwan). Ionic currents in GH_3_ or HL-1 cells were measured with an RK-400 operational patch-clamp amplifier (Bio-Logic, Claix, France) or an Axopclamp-2B amplifier (Molecular Devices, Sunnyvale, CA, USA), which was equipped with a Digidata 1440A device (Molecular Devices). Ionic currents were recorded in whole-cell or cell-attached configuration of the patch-clamp technique [23,24]. By using a PP-830 vertical puller (Narishige; Taiwan Instrument, Taipei, Taiwan) or a Flaming-Brown P97 horizontal puller (Sutter, Novato, CA, USA), the recording pipettes were pulled from Kimax-51 (#34500) borosilicate glass capillaries (Kimble; Dogger, New Taipei City, Taiwan), and they had tip resistances of 3–5 MΩ in situations when filled with internal pipette solutions stated above. All measurements were undertaken at room temperature (20–25 °C) on the stage of an inverted DM-II fluorescence microscope (Leica; Major Instruments, Kaohsiung, Taiwan). Data acquisition with varying voltage-clamp waveforms (i.e., analog-to-digital and digital-to-analog) was performed using the pClamp 10.7 software suite (Molecular Devices). The liquid junction potentials were zeroed immediately before seal formation was made, and the whole-cell data were corrected.

The signals were monitored and digitally stored on-line at 10 kHz in an ASUS ExpertBook laptop computer (P2451F; Yuan-Dai, Tainan, Taiwan). During the measurements, the Digidata 1440A was operated using pClamp 10.7 software run on Microsoft Windows 7 (Redmond, WA, USA). The laptop computer was placed on the top of an adjustable Cookskin stand (Ningbo, Zhejiang, China) to enable efficient operation during the measurements.

### 2.4. Whole-Cell Data Analyses

To determine concentration-dependent stimulation of apocynin on the transient (peak) or late I_Na_, we kept cells bathed in Ca^2+^-free Tyrode’s solution. During the measurements, we voltage-clamped the examined cell at −80 mV and the brief depolarizing pulse to −10 mV was applied to evoke I_Na_. The late I_Na_ in response to 100 μM *aPO* was taken as 100% and those (i.e., peak and late I_Na_) during exposure to different *aPO* concentrations (0.3–30 μM) were thereafter compared. The concentration-response data for stimulation of peak or late I_Na_ in pituitary GH_3_ cells were least-squares fitted to the Hill equation. That is,

(1) $$percentage\;decrease\left(\%\right)=\frac{E_{max}\times {\left[aPO\right]}^{n_{H}}}{EC_{50}^{n_{H}}+{\left[aPO\right]}^{n_{H}}}$$

In this equation, [*aPO*] is the *aPO* concentration used, *^n^_H_* the Hill coefficient, EC_50_ the concentration needed for a 50% inhibition of peak or late I_Na_, and *E_max_* the maximal stimulation of peak or late I_Na_ caused by the addition of *aPO*.*k*.

The stimulatory effect of *aPO* on I_Na_ is thought to be explained by a state-dependent activator that binds preferentially to the open state of the Na_V_ channel. From a simplifying assumption, the first-order binding scheme was given as follows:

(2)
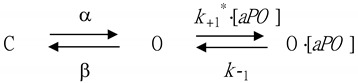

or

(3) $$\frac{dC}{dt}=O\times \beta -C\times \alpha$$

(4) $$\frac{dO}{dt}=C\times \alpha +O\cdot \left[aPO\right]\times k_{-1}-O\times \beta -O\times k_{+1}^{*}\cdot \left[aPO\right]$$

(5) $$\frac{d\left(O\cdot \left[aPO\right]\right)}{dt}=O\times k_{+1}^{*}\left[aPO\right]-O\cdot \left[aPO\right]\times k_{-1}$$

where [*aPO*] is the *aPO* concentration applied, and α or β the voltage-gated rate constant for the opening or closing of the Na_v_ channels, respectively. k*_+__1_ or *k*_−1_ represents the forward (i.e., on or bound) or reverse (i.e., off or un-bound) rate constant of *aPO*, respectively, while C, O, or O·[*aPO*] in each term denotes the closed (resting), open, or open-[*aPO*] state, respectively.

**Figure 1:**
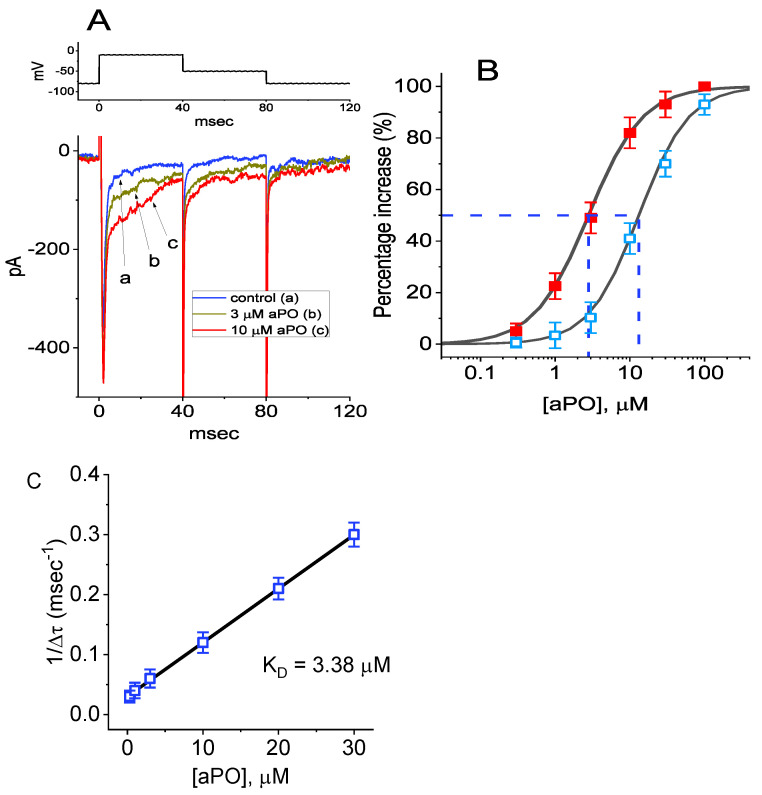
Effect of *aPO* on the peak and late components of voltage-gated Na^+^ current (I_Na_) identified in pituitary GH_3_ cells. These experiments were undertaken in cells bathed in Ca^2+^-free Tyrode’s solution containing 10 mM tetraethylammonium chloride (TEA), whereas the recording pipette was filled up with Cs^+^-enriched solution. (**A**) Representative I_Na_ traces activated by brief depolarizing pulse (indicated in the upper part). a: control (i.e., *aPO* was not present); b: 3 μM *aPO*; c: 10 μM *aPO*. (**B**) Concentration-dependent stimulation of *aPO* on peak or late I_Na_ (mean ± SEM; n = 8 for each point). The peak (□) or late (■) amplitude of the current was measured at the beginning or end of a 40-ms depolarizing pulse from −80 to −10 mV. Data analysis was performed by ANOVA-1 (*p* < 0.05). Each continuous line illustrates the goodness-of-fit to the Hill equation, as elaborated in Materials and Methods. The vertical broken line indicates the EC_50_ value required for 50% stimulation of the current (peak or late I_Na_). (**C**) The relationship of the reciprocal to the time constant (i.e., 1/∆τ) versus the *aPO* concentration was plotted (mean ± SEM; n = 7–11 for each point). From the binding scheme (indicated under Materials and Methods), the forward (*k*_+1_*) or backward (*k*_−1_) rate constant for *aPO*-accentuated I_Na_ in GH_3_ cells was computed to be 0.00898 ms^−1^μM^−1^ or 0.0303 ms^−1^, respectively.

Forward or backward rate constants, k*_+__1_ or *k*_−1_, were respectively determined from the time constants of current decay activated by the brief step depolarization from −80 to −10 mV. The time constants of I_Na_ inactivation were estimated by fitting the inactivation trajectory of each current trace with a double exponential curve (i.e., fast and slow components of current inactivation). These rate constants would be evaluated using the following equation:

(6) $$\frac{1}{\Delta \tau }=k_{+1}^{*}\times \left[aPO\right]+k_{-1}$$

where k*_+1_ or *k*_−1_, respectively, are ascribed from the slope or from the y-axis intercept at [*aPO*] = 0 of the linear regression in which the reciprocal time constant (i.e., 1/∆τ) versus varying *aPO* concentrations was interpolated. ∆τ indicates the difference in the slow component of current inactivation (τ_inact(S)_) obtained when the τ_inact(S)_ value during exposure to each concentration (0.03–30 μM) was subtracted from that in the presence of 100 μM *aPO* (Figure 1C).

The quasi-steady-state inactivation curve of peak I_Na_ with or without the *aPO* addition identified in GH_3_ cells was established on the basis of a simple Boltzmann distribution (or the Fermi–Dirac distribution):

(7) $$I=\frac{I_{max}}{1+e^{\frac{\left(V-V_{1/2}\right)qF}{RT}}}$$

where *I_max_* is the maximal peak I_Na_ in the absence or presence of 10 μM *aPO*; *V* the conditioning potential in mV; *V*_1/2_ the half-maximal inactivation in the relationship of the curve; *q* the apparent gating charge; *F* Faraday’s constant; R the universal gas constant; and *T* the absolute temperature.

### 2.5. Curve-Fitting Procedures and Statistical Analyses

Curve fitting (linear or non-linear (e.g., exponential or sigmoidal curve)) to various data sets was carried out with the goodness of fit by using various maneuvers, such as the Microsoft “Solver” function embedded in Excel 2019 (Microsoft) and 64-bit OriginPro^®^ 2016 program (OriginLab; Scientific Formosa, Kaohsiung, Taiwan). The data are presented as the mean ± standard error of the mean (SEM), with sample sizes (n) indicating the number of GH_3_ or HL-1 cells from which the data were collected. The Student’s *t*-test (paired or unpaired) and the analyses of variance (ANOVA-1 or ANOVA-2) with or without repeated measures followed by post-hoc Fisher’s least-significant different test were performed. The analyses were performed using SPSS version 20.0 (Asia Analytics, Taipei, Taiwan). A *p* value of less than 0.05 was considered to indicate the statistical difference.

## 3. Results

### 3.1. Effect of aPO on the Voltage-Gated Na+ Current (I_Na_) Recorded from Pituitary GH_3_ Cells

In the first stage of measurements, we kept cells immersed in a Ca^2+^-free Tyrode’s solution containing 0.5 mM CdCl_2_, the composition of which was stated in Materials and Methods, and we filled up the pipette by using the Cs^+^-containing solution. As the whole-cell configuration was firmly established, we voltage-clamped the tested cell at the level of −80 mV and a brief step depolarization to −10 mV was delivered to activate I_Na_ with a rapid activation and inactivation [23,25,26]. Of interest, one minute after cells were continually exposed to *aPO*, the peak amplitude of I_Na_ was progressively increased, and the concomitant inactivation time course of the current slowed (Figure 1A). In the presence of 10 μM *aPO*, the peak I_Na_ amplitude in response to rapid depolarizing pulse from −80 to −10 mV was significantly increased to 445 ± 31 pA (n = 9, *p* < 0.05) from a control value of 315 ± 22 pA. Additionally, the slow component of the inactivation time constant of I_Na_ activated by brief membrane depolarization was conceivably prolonged to 65.1 ± 10.2 ms (n = 9, *p* < 0.05) from a control value of 11.3 ± 2.3 ms (n = 9), although the fast component of the inactivation time constant did not differ significantly between absence and presence of *aPO*. After washout of *aPO*, the current amplitude was back to 306 ± 19 pA (n = 8, *p* < 0.05). Similarly, the deactivation time course of I_Na_ at −50 mV was prolonged in the presence of *aPO*.

The relationship between the *aPO* concentration and the peak or late component of I_Na_ was further analyzed and constructed in GH_3_ cells. Each cell was depolarized from −80 to −10 mV and current amplitudes at different concentrations (0.3–100 μM) of *aPO* were compared. As can be seen in Figure 1B, the application of *aPO* resulted in a concentration-dependent increase in peak or late I_Na_ activated by a short depolarizing pulse. The EC_50_ value for *aPO*-stimulated peak or late I_Na_ was 13.2 or 2.8 μM, respectively, and *aPO* at a concentration of 100 μM almost fully increased I_Na_. The data, therefore, reflect that *aPO* has a specific stimulatory action on I_Na_ in GH_3_ cells, and that the late component of I_Na_ was stimulated to a greater extent than the peak component of the current.

### 3.2. Evaluating aPO’s Time-Dependent Slowing of I_Na_ Inactivation

It needs to be mentioned that increasing *aPO* not only resulted in increased amplitude in the peak I_Na_ but also caused a clear and marked retardation in the magnitude of I_Na_ inactivation in response to rapid membrane depolarization. According to the first-order reaction scheme (indicated under Materials and Methods), the relationship between 1/∆τ and [*aPO*] turned out to be linear (Figure 1C). The forward and backward rate constants were estimated to be 0.00898 ms^−1^μM^−1^ or 0.0303 ms^−1^, respectively; thereafter, the apparent dissociation constant (i.e., K_D_ = k_−1_/k_+1_*) for the binding of *aPO* to the Na_v_ channels was consequently yielded to be 3.4 μM, a value which was noticeably close to the estimated EC_50_ value for *aPO*-mediated stimulation of late I_Na_ determined from the concentration-response curve (Figure 1B).

### 3.3. Effect of aPO on the Current-Voltage (I-V) Relationship or Steady-State Inactivation Curve of I_Na_

**Figure 2:**
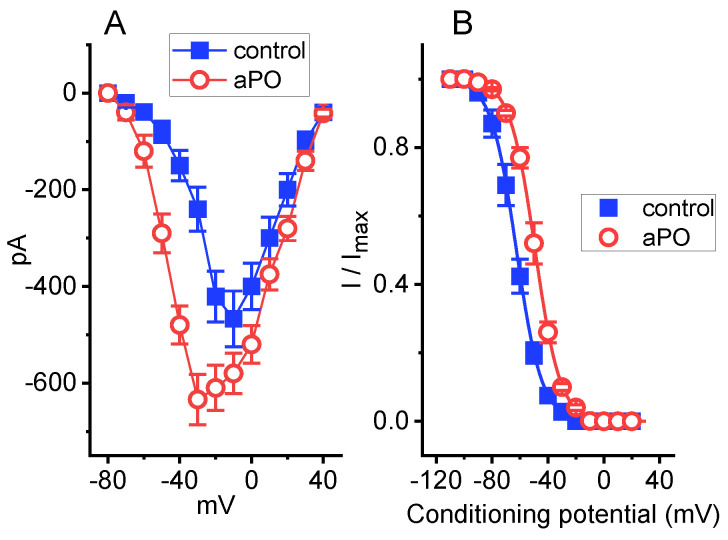
Stimulatory effect of *aPO* on averaged current–voltage (I-V) relationship (**A**) and steady-state inactivation curve (**B**) of I_Na_ present in GH_3_ cells. Cells were kept bathed in Ca^2+^-free Tyrode’s solution containing 10 mM TEA. (**A**) Averaged I-V relationships of I_Na_ in the absence (■) and presence (**○**) of 10 μM *aPO* (mean ± SEM; n = 8 for each point). The examined cell was held at −80 mV and the 40-ms voltage pulse ranging from −80 to +40 mV in 10-mV steps was delivered to it. The statistical analyses were undertaken by ANOVA-2 for repeated measures, *p* (factor 1, groups among data ken at different level of voltages) < 0.05, *p* (factor 2, groups between the absence and presence of *aPO*) < 0.05, *p* (interaction) < 0.05, followed by post-hoc Fisher’s least-significant difference test, *p* < 0.05). (**B**) Effect of *aPO* on the steady-state inactivation curve of I_Na_ taken without (■) or with (**○**) the addition of 10 μM *aPO*. In these experiments, the conditioning voltage pulses with a duration of 40 ms to various membrane potentials between −120 and +20 mV were applied from a holding potential of −80 mV. Following each conditioning potential, a test pulse to −10 mV with a duration of 40 ms was delivered to activate I_Na._ The normalized amplitude of I_Na_ (I/I_max_) was constructed against the conditioning potential and the sigmoidal curves were optimally fitted by the Boltzmann equation (indicated under Materials and Methods). Each point represents the mean ± SEM (n = 7). The statistical analyses were undertaken by ANOVA-2 for repeated measures, *p* (factor 1, groups among data ken at different level of conditioning potentials) < 0.05, *p* (factor 2, groups between the absence and presence of *aPO*) < 0.05, *p* (interaction) < 0.05, followed by post-hoc Fisher’s least-significant difference test, *p* < 0.05).

We continued to examine the stimulatory effect of *aPO* at different membrane potential, and an I-V relationship of I_Na_ without or with the *aPO* addition was constructed. As depicted in Figure 2A, the I-V relationship of I_Na_ was shifted slightly to more negative potentials during cell exposure to *aPO* (10 μM). Additionally, the stimulatory effect of *aPO* on the steady-state inactivation curve of I_Na_ was further characterized (Figure 2B). In this stage of experiments, a 40-ms conditioning pulse to various membrane potentials (from −120 to +20 mV in 10-mV steps) was delivered to precede the test pulse (40 ms in duration) to −10 mV from a holding potential of −80 mV. Under this experimental protocol, the relationship between the conditioning potentials and the normalized amplitudes of I_Na_ with or without the addition of *aPO* (10 μM) was constructed and properly fitted to a Boltzmann type sigmoidal function (indicated under Materials and Methods) by using a non-linear regression analysis. In the absence and presence of 10 μM *aPO*, the V_1/2_ value was noticed to differ significantly (−62.6 ± 1.3 mV (in the control) versus −49.2 ± 1.4 mV (in the presence of *aPO*); n = 7, *p* < 0.05); in contrast, the value of q (apparent gating charge) did not differ significantly (2.79 ± 0.12 e (in the control) versus 2.82 ± 0.13 e (in the presence of *aPO*); n = 7, *p* > 0.05). Therefore, cell exposure to *aPO* not only increased the maximal conductance of I_Na_, but also shifted the inactivation curve to the rightward direction by approximately 13 mV. However, we found no evident change in the gating charge of the inactivation curve during cell exposure to *aPO*. As such, it is reasonable to assume that the steady-state I_Na_ inactivation curve in the presence of this compound was shifted rightward, with no clear adjustment in the gating charge of this curve.

### 3.4. Effect of aPO on the Recovery from I_Na_ Inactivation by Using Two-Step Voltage Protocol

**Figure 3:**
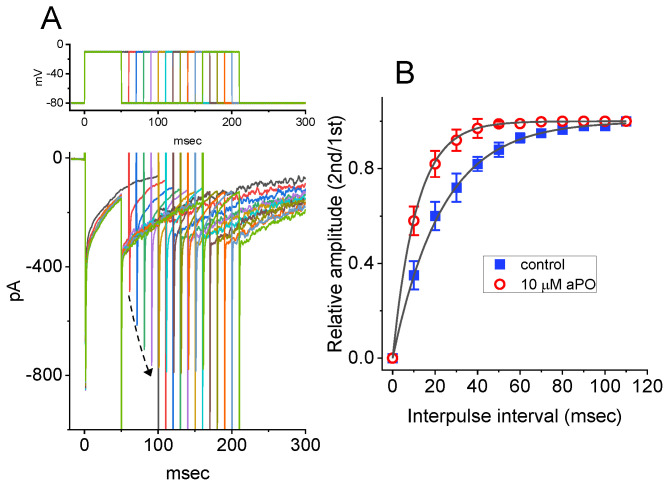
Effect of *aPO* on the time course of recovery from I_Na_ inactivation. The cell tested was depolarized from −80 to −10 mV with a duration of 50 ms, and voltage-clamp commands with varying durations of interpulse interval (i.e., the interval between the first and second pulses) were applied to it. (**A**) Superimposed I_Na_ traces in the presence of 10 μM *aPO*. The upper part shows the voltage protocol applied. The dashed arrow indicates the trajectory of current inactivation elicited by different durations of interpulse pulse. (**B**) Effect of *aPO* on the time course of recovery from current inactivation, as the cells examined were depolarized from −80 to −10 mV. ■: control; **○**: *aPO* (10 μM). Each smooth line was optimally fitted by a single-exponential function. The relative amplitude denotes that the peak I_Na_ taken at the second pulse is divided by that at the first one. Each point represents the mean ± SEM (n = 8). The statistical analyses were undertaken by ANOVA-2 for repeated measures, *p* (factor 1, groups among data ken at different interpulse intervals) < 0.05, *p* (factor 2, groups between the absence and presence of *aPO*) < 0.05, *p* (interaction) < 0.05, followed by post-hoc Fisher’s least-significant difference test, *p* < 0.05).

We then examined whether the presence of *aPO* produces any effect on the recovery of I_Na_ from inactivation. In a two-step voltage protocol, a 50-ms conditioning step to −10 mV inactivated most of the current, and the recovery from current inactivation at the holding potential of −80 mV was examined at different times with a test step (−10 mV, 50 ms), as demonstrated in Figure 3A,B. In the control period (i.e., *aPO* was not present), the peak amplitude of I_Na_ nearly completely recovered from inactivation when the interpulse duration was set at 100 ms. The time constant course of recovery from current inactivation in the absence or presence of *aPO* (10 μM) was least-squares fitted to a single-exponential function with a time constant of 23.3 ± 1.1 or 11.3 ± 0.9 ms (n = 8, *p* < 0.05), respectively. These experimental observations indicate that cell exposure to *aPO* produces a significant shortening in the recovery from inactivation of I_Na_ in GH_3_ cells.

### 3.5. Comparison among Effects of aPO, Tefluthrin (Tef), Tef Plus aPO, aPO Plus Rufinamide (RFM), and aPO Plus Ranolazine (Ran) on the Peak Amplitude of I_Na_

**Figure 4:**
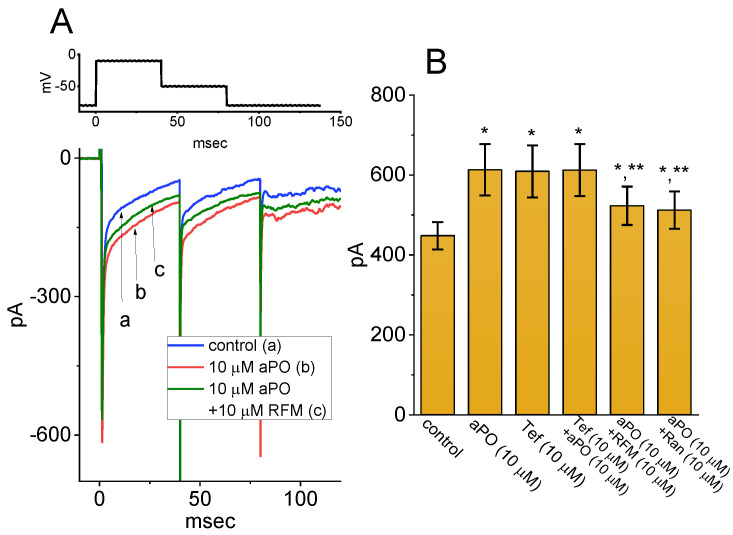
Effect of *aPO*, tefluthrin (Tef), Tef plus *aPO*, *aPO* plus rufinamide (RFM), and *aPO* plus ranolazine (Ran) on peak amplitude of I_Na_ identified in GH_3_ cells. (**A**) Representative I_Na_ traces activated by depolarizing pulse (as indicated in the upper part). a: control; b: 10 μM *aPO*; c: 10 μM *aPO* plus 10 μM RFM. (**B**) Summary bar graph showing effect of *aPO*, Tef, Tef plus *aPO*, *aPO* plus RFM, and *aPO* plus Ran on peak I_Na_ (mean ± SEM; n = 8–10 for each bar). The number of the control group is 10, while those in other groups are 8. Data analysis was performed by ANOVA-1 (*p* < 0.05). * Significantly different from control (*p* < 0.05) and ** significantly different from *aPO* (10 μM) alone group (*p* < 0.05).

Tef, a type-I pyrethroid insecticide, was reported to be an activator of I_Na_ [23,24,25,27], Ran is recognized as a late I_Na_ blocker as well as an inhibitor of NOX activity [26,28,29,30], and RFM, known to be an antiepileptic agent, was previously demonstrated to perturb I_Na_ inactivation [31,32]. For these reasons, we further examined and then compared the effects of these agents on peak I_Na_ identified in GH_3_ cells. As demonstrated in Figure 4, in accordance with previous studies [23], one minute after Tef (10 μM) was applied, it was effective in stimulating peak I_Na_. However, in the continued presence of Tef for two minutes, one minute after further addition of 10 μM *aPO*, peak I_Na_ was not increased further. In addition, as cells were continually exposed to 10 μM *aPO*, subsequent application of 10 μM RFM or 10 μM Ran was able to attenuate *aPO*-induced stimulation of I_Na_ one minute later. The results imply that *aPO* and Tef share a similarity to their stimulation of I_Na_, and that further addition of RFM or Ran is effective in attenuating *aPO*-stimulated I_Na_ in GH_3_ cells.

### 3.6. Stimulatory Action of aPO on I_Na_ in Methylglyoxal- (MeG-) or Superoxide Dismutase- (SOD-) Treated Cells

**Figure 5:**
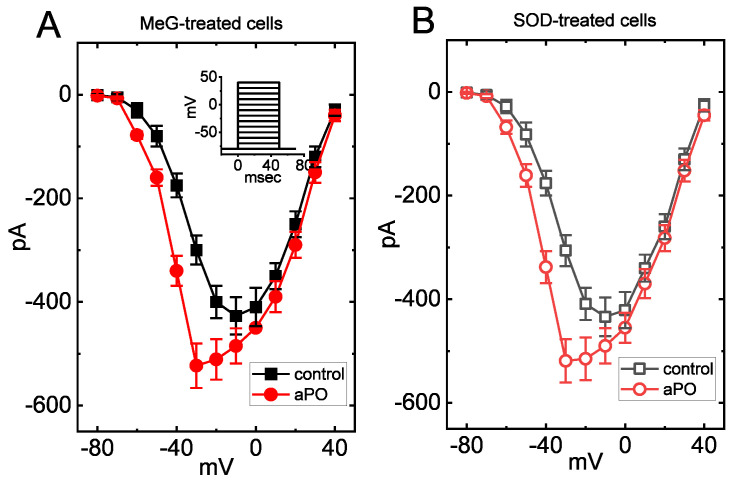
Stimulatory effect of *aPO* on averaged I-V relationship of I_Na_ in GH_3_ cells treated with methylglyoxal (MeG) (**A**) or with superoxide dismutase (SOD) (**B**). GH_3_ cells were preincubated with 10 μM MeG for 6 h. Cells were bathed in Ca^2+^-free Tyrode’s solution and the pipette was filled up with Cs^+^-containing solution. The cell tested was maintained at −80 mV and the depolarizing pulses ranging between −80 and +40 mV were thereafter delivered to it. Each point represents the mean ± SEM (n = 7). Inset denotes the voltage-clamp protocol used. ■ or □: control; ●or ○: *aPO* (10 μM). Noticeably, in MeG- or SOD-treated cells, the stimulatory effect of *aPO* on the overall I-V relationships of peak I_Na_ was altered little. The statistical analyses were undertaken by ANOVA-2 for repeated measures, *p* (factor 1, groups among data taken at different levels of voltages) < 0.05, *p* (factor 2, groups between the absence and presence of *aPO*) < 0.05, *p* (interaction) < 0.05, followed by post-hoc Fisher’s least-significant difference test, *p* < 0.05).

One would expect that the effect of *aPO* on I_Na_ is engaged in either its inhibition of NOX activity or the reduction in the production of reactive oxygen species. The expression of NOX was previously reported to be distributed in pituitary cells [14,15]. As such, the effect of *aPO* on I_Na_ was assessed in cells preincubated with MeG or SOD for 6 h. MeG was previously recognized to be a substrate for NOX activity [33,34,35], while SOD, an antioxidative enzyme, was reported to reduce the production of reactive oxygen species [36]. However, in GH_3_ cells preincubated with MeG for 6 h, the I-V relationship of peak I_Na_ with or without addition of *aPO* is illustrated in Figure 5. For example, in cells pretreated with MeG (10 μM), *aPO* (10 μM) could significantly increase the amplitude of I_Na_ measured at the level of −20 mV from 401 ± 31 to 511 ± 39 pA (n = 7, *p* < 0.05). Likewise, in SOD-preincubated cells, the addition of *aPO* (10 μM) increased I_Na_ amplitude at −20 mV from 409 ± 31 to 515 ± 41 pA (n = 7, *p* < 0.05). Therefore, these results allowed us to suggest that the stimulatory effect of *aPO* on I_Na_ that we obtained in these cells is unlikely to be due to changes in either the production of reactive oxygen species or cytosolic NOX activity.

### 3.7. Effect of aPO on the Amplitude and Voltage-Dependent Hysteresis (Vhys) of Persistent Na^+^ (I_Na(P)_)

**Figure 6:**
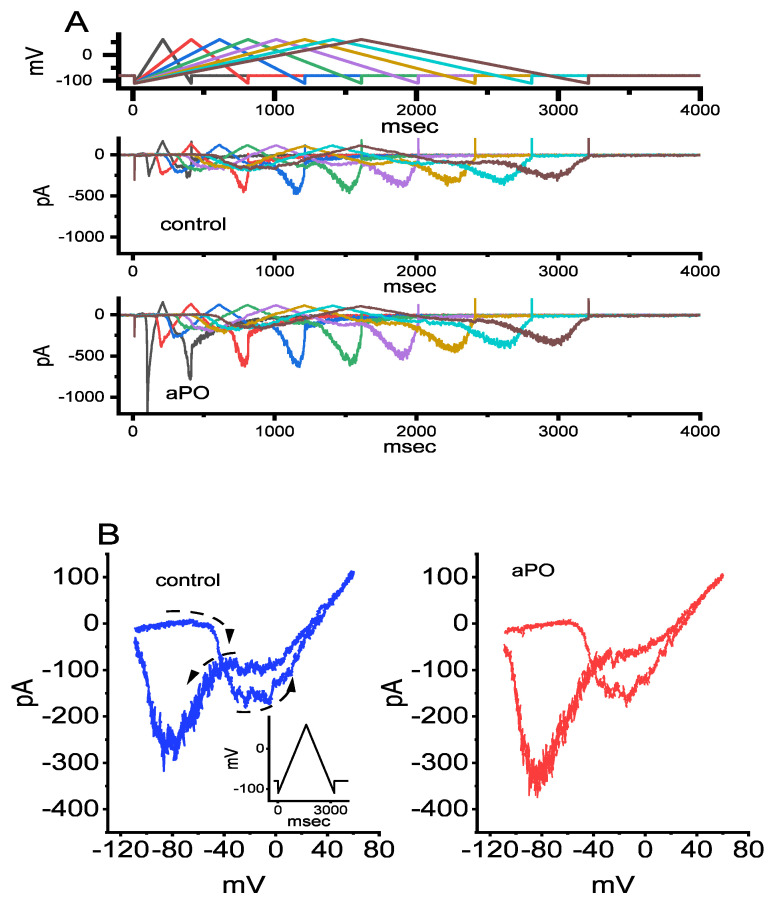

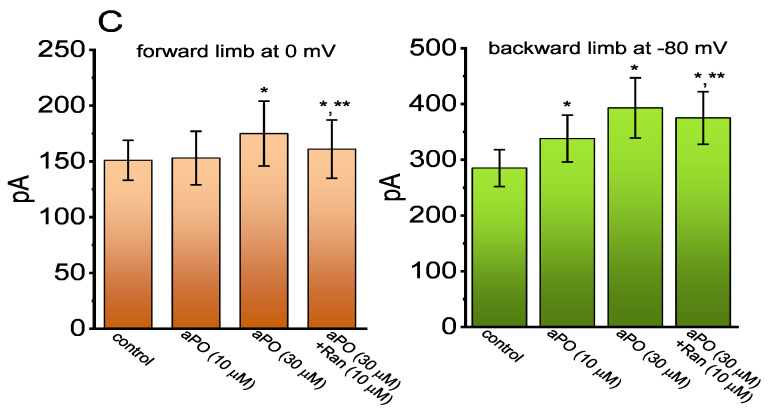
Effect of *aPO* on voltage-dependent hysteresis (Vhys) of persistent I_Na_ (I_Na(P)_) activated by isosceles-triangular ramp pulses with varying ramp duration in GH_3_ cells. In this series of whole-cell current recordings, we voltage-clamped the tested cell at −80 mV and the isosceles-triangular ramp voltage with varying duration of 0.4 to 3.2 s (i.e., ramp speed of ±0.1 to 0.8 mV/ms) to activate I_Na(P)_ in response to the forward (i.e., ascending from −110 to +50 mV) and backward (descending from +50 to −110 mV) that was thereafter applied to it. (**A**) Representative I_Na(P)_ traces obtained in the control period (upper, *aPO* was not present), and during cell exposure to 10 μM *aPO* (lower). The uppermost part shows varying durations of isosceles-triangular ramp pulse applied. Of notice, the presence of *aPO* can augment the I_Na(P)_ amplitude elicited by the upsloping and downsloping limbs of the triangular ramp. (**B**) Representative instantaneous I-V relation of I_Na(P)_ in response to isosceles-triangular ramp pulse (the voltage between −100 and +50 mV) with a duration of 3.2 s (as indicated in the left side of panel (**B**)). Current trace in the left side is control, while that in the right side was acquired from the presence of 10 μM *aPO*. The dashed arrows in the left side show the direction of I_Na(P)_ trajectory in which time passes during the elicitation by the upright isosceles-triangular ramp pulse. Of interest, a striking figure-of-eight (or infinity-shaped: ∞) exists in the Vhys trajectory responding to the triangular ramp. (**C**) Summary bar graph demonstrating the effect of *aPO* and *aPO* plus Ran on I_Na(P)_ amplitude activated by the upsloping and downsloping limbs of 3.2-s triangular ramp pulse (mean ± SEM; n = 8 for each bar). Current amplitudes in the left side were taken at the level of 0 mV in situations where the 1.6-s ascending (upsloping) end of the triangular pulse was delivered to elicit I_Na(P)_ (i.e., high-threshold I_Na(P)_, while those in the right side (i.e., low-threshold I_Na(P)_) was at −80 mV during the descending (downsloping) end of the pulse. Current amplitude measured is illustrated in the absolute value. Data analyses were performed by ANOVA-1 (*p* < 0.05). * Significantly different from controls (*p* < 0.05) and ** significantly different from *aPO* (30 μM) alone groups (*p* < 0.05).

The Vhys behavior residing in varying types of ion channels (i.e., the difference in current trajectory in response to the upsloping and the downsloping voltages) is currently a subject of extensive research, including Na_V_ channels [24,37,38]. We next examined whether or how the presence of *aPO* is able to modify I_Na(P)_ Vhys activated in response to the upright isosceles-triangular ramp pulse in GH_3_ cells. In this stage of our whole-cell current recordings, the tested cell was voltage-clamped at the level of −80 mV and we then applied it with a set of isosceles-triangular ramp pulses ranging between −110 and +50 mV (with a height of 160 mV) of varying ramp duration at a rate of 0.05 Hz through digital-to-analog conversion (Figure 6A). Consistent with previous observations [24,26], the amplitude of I_Na(P)_ in response to such upright triangular ramp voltage was noticed to display a striking figure-of-eight Vhys (i.e., ∞) in the instantaneous I-V relationship of I_Na(P)_ with two distinct peaks, i.e., low and high threshold I_Na(P)_. Alternatively, there is an initial counterclockwise direction, which time goes by, in current trajectory (i.e., high-threshold loop with a peak at −0 mV) activated by the upsloping limb, and following the downsloping limb, a clockwise direction (i.e., low-threshold loop with a peak at −80 mV) ensued (Figure 6B). Of particular interest, one minute after GH_3_ cells were exposed to 30 μM *aPO* alone, the amplitude of I_Na(P)_ at high or low threshold respectively activated by the upsloping triangular ramp voltage (forward or ascending) or downsloping (backward or descending) limb of upright triangular ramp voltage was increased. The augmentation of low-threshold I_Na(P)_ produced by 30 μM *aPO* was observed to be greater than that in the high-threshold one (Figure 6C), for example, as the isosceles-triangular ramp pulse with a duration of 3.2 s (or ramp speed of ±0.1 mV/ms). In the presence of 30 μM *aPO*, the peak I_Na(P)_ amplitude measured at the level of −0 mV (i.e., high-threshold I_Na(P)_) during the ascending phase of triangular ramp pulse was significantly raised to 175 ± 29 pA (n = 8, *p* < 0.05) from a control value (measured at the isopotential level) of 151 ± 18 pA (n = 8). Meanwhile, during cell exposure to 30 μM *aPO*, the peak I_Na(P)_ amplitude measured at −80 mV during the descending phase of such a ramp concurrently increased from 285 ± 33 to 393 ± 54 pA (n = 8, *p* < 0.05). Alternatively, the subsequent application of 10 μM Ran, but still in the continued presence of 30 μM *aPO*, was able to attenuate the *aPO*-mediated increase of I_Na(P)_ taken at either high or low threshold amplitude in the Vhys loop. These observations, therefore, enabled us to indicate that the Vhys strength of I_Na(P)_ activated by isosceles-triangular ramp pulses of varying ramp duration observed in GH_3_ cells was enhanced in the presence of *aPO* (Figure 6B,C).

### 3.8. Effect of aPO on Erg-Mediated K^+^ Current (I_K(erg)_) in GH_3_ Cells

**Figure 7:**
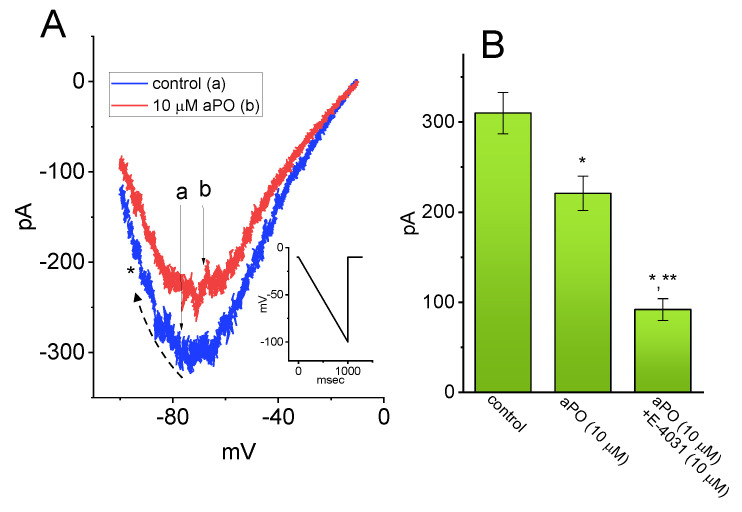
Effect of *aPO* on erg-mediated K+ current (I_K(erg)_) in GH_3_ cells. The experiments were undertaken in cells that were bathed in high-K^+^, Ca^2+^-free solution containing 1 μM tetrodotoxin (TTX), and the recording pipette was filled up with K^+^-containing internal solution. (**A**) Representative I_K(erg)_ traces obtained in the control (a) and during cell exposure to 10 μM *aPO* (b). The examined cell was held at −10 mV and a downsloping ramp from −10 to −100 mV with a duration of 1 s (indicated in the inset) was applied to it. The dashed arrow indicates the direction of current trajectory in which time passes, while the asterisk shows the inwardly-rectifying property of I_K(erg)_. (**B**) Summary bar graph showing effect of *aPO* and *aPO* plus E-4031 on the amplitude of I_K(erg)_ (mean ± SEM; n = 8 for each bar). Current amplitude (i.e., peak I_K(erg)_ amplitude) was measured at the level of −70 mV. Data analyses were performed by ANOVA-1 (*p* < 0.05). * Significantly different from control (*p* < 0.05) and ** significantly different from the *aPO* (10 μM) alone group (*p* < 0.05).

Earlier studies have demonstrated that telmisartan, an activator of I_Na_, can be effective in inhibiting I_K(erg)_ [22]. For this reason, we decided to investigate whether *aPO* exercises any perturbations on I_K(erg)_. The biophysical and pharmacological properties of I_K(erg)_ in GH_3_ cells have been previously reported [22,39,40,41]. In these whole-cell experiments, we bathed cells in high-K^+^, Ca^2+^-free solution, and the recording pipette was filled up with K^+^-containing solution. The composition of these solutions was detailed under Materials and Methods. The examined cell was voltage-clamped at −10 mV and the linear downsloping ramp pulse from −10 to −100 mV with a duration of 1 s was applied to it. As shown in Figure 7, the addition of 10 μM *aPO* resulted in a progressive decline in the amplitude of deactivating I_K(erg)_ in response to such a downsloping hyperpolarizing ramp. However, in the continued presence of *aPO*, further application of E-4031, an inhibitor of I_K(erg)_, was able to decease the current amplitude further. Therefore, unlike I_Na_ induced by *aPO*, I_K(erg)_ residing in these cells was subject to being inhibited by its presence.

### 3.9. Effect of aPO on I_Na_ Recorded from Murine HL-1 Cardiomyocytes

**Figure 8:**
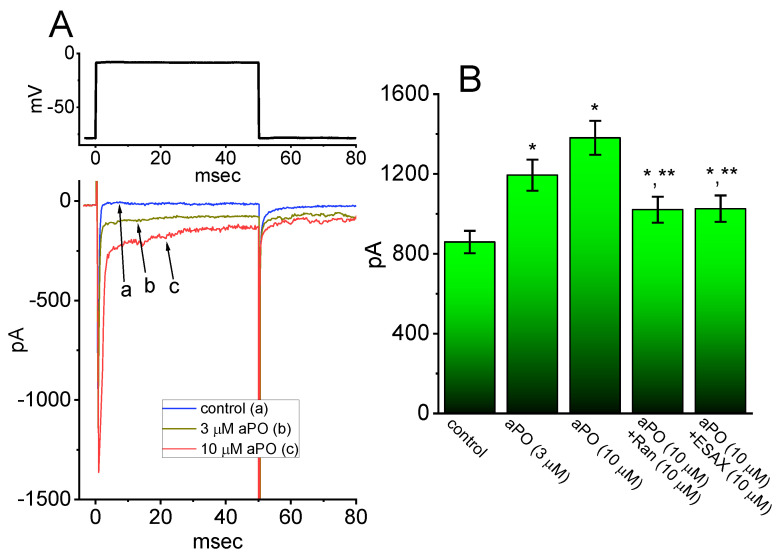
Effect of *aPO* on depolarization-activated I_Na_ present in HL-1 cardiomyocytes. In this set of experiments, we kept cells immersed in Ca^2+^-free Tyrode’s solution and the electrode was filled with Cs^+^-enriched solution. When whole-cell configuration was established, we voltage-clamped the cell at −80 mV and the brief depolarization to −10 mV was delivered to it. (**A**) Representative I_Na_ traces activated by depolarizing command pulse (indicated in the upper part). a: control; b: 3 μM *aPO*; c: 10 μM *aPO*. (**B**) Summary bar graph showing effects of *aPO*, *aPO* plus ranolazine (Ran), and *aPO* plus esaxerenone (ESAX) on peak amplitude of I_Na_ in HL-1 heart cells (mean ± SEM; n = 8 for each bar). Current amplitude was measured at the beginning of 50-ms depolarizing pulses from −80 to −10 mV. Data analyses were performed by ANOVA-1 (*p* < 0.05). * Significantly different from control (*p* < 0.05) and ** Significantly different *aPO* (10 μM) alone group (*p* < 0.05).

*aPO* was previously demonstrated to be a chemo-preventive agent for cardiovascular disorders though the inhibition of NOX activity [35,42,43,44]. In another set of experiments, we tested whether I_Na_ inherently in heart cells (i.e., HL-1 cardiomyocytes) could still be modified by the presence of *aPO*. The preparation of these cells was described above under Materials and Methods. Cells were kept bathed in Ca^2+^-free Tyrode’s solution in which 10 mM TEA was included, and the pipette was filled with Cs^+^-enriched solution. Noticeably, as HL-1 cells were continually exposed to *aPO* at a concentration of 3 or 10 μM, the amplitude of peak I_Na_ activated by 50-ms depolarizing pulses from −80 to −10 mV was increased; concomitantly, progressive slowing of the inactivation time course of the current was seen (Figure 8A,B). For example, cell exposure to 10 μM *aPO* resulted in a conceivable increase of peak I_Na_ from 859 ± 56 to 1381 ± 85 pA (n = 8, *p* < 0.05); concomitantly, the τ_inact(S)_ value was significantly raised to 56.3 ± 7.1 ms (n = 8, *p* < 0.05) from a control value of 7.1 ± 1.4 ms. After washout of *aPO* (i.e., *aPO* was removed, but cells were still exposed to Ca^2+^-free Tyrode’s solution containing 10 mM TEA), current amplitude returned 892 ± 58 pA (n = 8, *p* < 0.05). Alternatively, in the continued presence of *aPO* (10 μM), further application of either ranolazine (Ran, 10 μM) or esaxerenone (ESAX, 10 μM) was noticed to attenuate *aPO*-mediated stimulation of I_Na_ (Figure 8B). Like Ran. ESAX was recently reported to inhibit I_Na_ [24]. Therefore, consistent to some extent with the observations done in GH_3_ cells, the results reflect the effectiveness of *aPO* in stimulating I_Na_ in response to the rapid depolarizing step in HL-1 cells.

## 4. Discussion

The distinctive findings in the present study are that (a) GH_3_-cell exposure to *aPO* could increase I_Na_ in a concentration, time-, state-, and Vhys-dependent fashion; (b) this agent resulted in the differential stimulation of peak or late amplitude of I_Na_ activated by abrupt step depolarization with aneffective EC_50_ value of 13.2 or 2.8 μM, respectively; (c) *aPO* mildly shifted the I-V curve of I_Na_ towards the depolarized potentials (i.e., a leftward shift), and it also made a rightward shift in the steady-state inactivation curve of the current towards the right side with no changes in the gating charge of the curve; (d) the recovery of the I_Na_ block was enhanced in its presence; (e) subsequent addition of rufinamide (RFM) or ranolazine (Ran) counteracted *aPO*-accentuated I_Na_; (f) the stimulatory effect of *aPO* on I_Na_ remained unaltered in cells preincubated with MeG or SOD; (g) *aPO* was capable of increasing the high- or low-threshold amplitude of I_Na(P)_ elicited by the isosceles-triangular ramp at either upsloping (ascending) or downsloping (descending) limb, respectively; (h) the *aPO* presence mildly decreased the amplitude of I_K(erg)_ activated by the downsloping ramp pulse; and (i) the exposure to *aPO* was effective at increasing the amplitude and inactivation time constant of I_Na_ in HL-1 atrial cardiomyocytes. Collectively, the present results allow us to reflect that *aPO*-stimulated changes in the amplitude, gating, and Vhys behavior of I_Na_ appear to be unlinked to and upstream of its inhibitory action on NOX activity, and that it would participate in the adjustments of varying functional activities in electrically excitable cells (e.g., GH_3_ or HL-1 cells), presuming that similar in vivo findings exist.

From the overall I-V relationship of I_Na_ demonstrated here, there was a slight shift toward more negative potential in the presence of *aPO*. The steady-state inactivation curve of I_Na_ in its presence of *aPO* was also shifted to a rightward direction with no apparent change in the gating charge of the curve. The increased recovery of the I_Na_ block was demonstrated in its presence. As a result, the window current of I_Na_ in GH_3_ cells was expected to be increased during cell exposure to *aPO*. Such a small molecule may have higher affinity to the open/inactivated state than to the resting (closed) state residing in the Nav channels, despite the detailed ionic mechanism of its stimulatory action on the channel remaining elusive.

Several lines of clear evidence have been demonstrated to indicate that *aPO* can inhibit NOX activity and decrease the production of superoxide oxide [2,3,4,16]. Pituitary cells have been previously demonstrated to be expressed in the activity of NOX [14,15,16]. As such, the question arises as to whether the stimulatory effect of *aPO* on I_Na_ observed in GH_3_ cells may actually result from either the reduction of NOX activity or the decreased level of superoxide anions [15,16]. However, this notion appears to be difficult to reconcile with the present observations disclosing that in GH_3_ cells preincubated with MeG or SOD, the stimulatory effect of *aPO* on I_Na_ was indeed observed to remain effective. It is also noted that *aPO* can mildly inhibit the amplitude of I_K(erg)_. Therefore, under our experimental conditions, the stimulation of I_Na_ caused by *aPO* tends to emerge in a manner largely independent of its inhibitory effect on NOX activity; hence, the *aPO* molecule can exert an interaction at binding site(s) inherently existing on Na_v_ channels.

Perhaps more important than the issue of the magnitude of the *aPO*-induced increase in I_Na_ is that we observed the non-linear Vhys of I_Na(P)_ in the control period (i.e., *aPO* was not present) and during cell exposure to *aPO* or *aPO* plus Ran, by use of the upright isosceles-triangular ramp voltage command of varying duration through digital-to-analog conversion. In particular, when cells were exposed to *aPO*, the peak I_Na(p)_ activated by the forward (ascending or upsloping) end of the triangular ramp of varying duration was observed to be elevated, particularly at the peak level of 0 mV, whereas the I_Na(P)_ amplitude at the backward (descending or downsloping) end was increased at the peak level of −80 mV. In this respect, the figure-of-eight (i.e., infinity-shaped: ∞) configuration in the Vhys loop activated by the triangular ramp pulse was evidently demonstrated (Figure 6A,B). Additionally, there appeared to be two types of Vhys loops, that is, a low-threshold loop with a peak at −80 mV (i.e., activating at a voltage range near the resting potential) and a high-threshold loop with a peak at 0 mV (i.e., activating at a voltage range near the maximal I_Na_ elicited by rectangular depolarizing step. The presence of *aPO* was capable of enhancing the Vhys strength of I_Na(P)_ and, in its continued presence, further addition of Ran attenuated *aPO*-increased Vhys loop of the current. In this scenario, findings from the present observations disclosed that the triangular pulse-induced I_Na(P)_ was detected to undergo striking Vhys change (i.e., initial counterclockwise direction followed by clockwise one) in the voltage-dependence and that such Vhys loops were subject to enhancement by the presence of *aPO*.

Pertinent to the stimulatory effect of *aPO* on I_Na_ is that in this study, due to its effectiveness in increasing the Vhys magnitude of I_Na(P)_, the voltage-dependent movement of the S4 segment residing in Na_V_ channels is probably perturbed by this agent; consequently, the coupling of the pore domain to the voltage-sensor domain, which the S1–S4 segments comprise, tended to be facilitated [45,46]. Indeed, the voltage sensor energetically coupled to channel activation, which might be influenced by the *aPO* molecule, is supposed to be a conformationally flexible region of the Na_V_-channel protein. Therefore, these findings can be interpreted to mean either that such I_Na(P)_, particularly during exposure to *aPO*, is intrinsically and dynamically endowed with “memory” of previous (or past) events, which is encoded in the conformational (or metastable) states of the Nav-channel protein, or that there is a mode shift of channel kinetics occurring regarding the voltage sensitivity of gating charge movement, which relies on the previous state (or conformation) of the Na_v_ channel [37,38]. Such a striking type of Vhys natively in Na_V_ channels would potentially play substantial roles in interfering with electrical behavior, Na^+^ overload, and hormonal sretion in varying types of excitable cells [37]. It is also worth pointing out that the subsequent addition of Ran, still in the continued presence of *aPO*, did produce a considerable reduction in the *aPO*-mediated increase in Vhys responding to triangular ramp voltage.

From pharmacokinetic studies in mice [47], following intravenous injection of *aPO* (5 mg/kg), the peak plasma *aPO* level was detected at 1 min to reach around 5500 ng/mL (or 33.1 μM). Additionally, *aPO* was reportedly a selective inhibitor of NOX2 activity with an effective IC_50_ of 10 μM [48]. According to the data of Figure 1, the IC_50_ value required for the *aPO*-stimulated peak or late I_Na_ was 13.2 or 2.8 μM, respectively, while the K_D_ value estimated on the basis of minimal reaction scheme was 3.4 μM. It is reasonable to assume, therefore, that *aPO*-induced changes in the amplitude, gating or Vhys behavior of I_Na_ presented herein could be highly achievable and of pharmacological relevance.

On the basis of the present experimental observations, despite the inhibitory effect on NOX activity [2,3,4], our results strongly suggest that the stimulatory actions of *aPO* on transmembrane ionic currents, particularly on Na_V_ channels, tends to be direct obligate mechanisms. Pyrethroids (e.g., permethrin and cypermethrin), known to activate I_Na_, have also been reported to disrupt NOX activity in brain tissue (striatum) [49]. Therefore, through ionic mechanisms shown herein, pyrethroids or other structurally similar compounds are able to adjust the functional activities of varying types of neuroendocrine or endocrine cells, or heart cells, if similar in vivo results exist [6,7,11,12,13,50]. To this end, the overall findings from our study highlight an important alternative aspect that has to be taken into account, inasmuch as there is the beneficial or ameliorating effect of *aPO* in various pathologic disorders, such as inflammatory or neurodegenerative diseases, and heart failure [1,3,6,7,9,10,11,12,13,16,42].

## Data Availability

The original data is available upon reasonable request to the corresponding author.

## Supplementary Materials

The details of cell preparation in Materials and Methods were mentioned in Supplementary Material which is available online https://www.mdpi.com/article/10.3390/biomedicines9091146/s1.

## Abbreviations

AP, action potential; *aPO* (apocynin, 4′-Hydroxy-3′-methoxyacetophenone); EC_50_, concentration required for 50% stimulation; erg, ether-à-go-go-related gene; ESAX, esaxerenone; I-V, current versus voltage; I_K(erg)_, erg-mediated K^+^ current; I_Na_, voltage-gated Na^+^ current; I_Na(P)_, persistent Na+ current; K_D_, dissociation constant; MeG, methylglyoxal; NADPH oxidase, nicotinamide adenine dinucleotide phosphate oxidase; Nav channel; voltage-gated Na^+^ channel; NADPH, nicotinamide adenine dinucleotide phosphate; NOX, NADPH oxidase; Vhys, voltage-dependent hysteresis; Ran, ranolazine; RFM, rufinamide; SEM, standard error of the mean; SOD, superoxide dismutase; τ_inact(S)_, slow component of inactivation time constant; TEA, tetraethylammonium chloride; Tef, tefluthrin; TTX, tetrodotoxin.
