# Supplementary material for: Effective Accentuation of Voltage-Gated Sodium Current Caused by Apocynin (4′-Hydroxy-3′-methoxyacetophenone), a Known NADPH-Oxidase Inhibitor
Source: Biomedicines. 2021 Sep 3;9(9):1146. doi: 10.3390/biomedicines9091146 (PMC8464932; doi:10.3390/biomedicines9091146)
Supplement: Supplementary file 1 [file biomedicines-09-01146-s001.zip › biomedicines-1351792-supplementary.pdf]

Supplemental materials

Cell preparations

GH_3 (a cell line from an anterior pituitary adenoma carried in a 7-month-old female Wistar-Furth rat) cells were acquired from the Bioresource Collection and Research Center (BCRC number: 60015; Hsinchu, Taiwan) [1]. They were cultured in Ham's F-12 medium (HyClone™) supplemented with 15% horse serum (v/v), 2.5% fetal calf serum (v/v), and 2 mM L-glutamine. The HL-1 atrial cell line was derived from the AT-1 mouse atrial cardiomyocyte tumor lineage, which was originally obtained from Louisiana State University in New Orleans, LA. HL-1 cells were maintained in Claycomb medium (Sigma-Aldrich) supplemented with 10% fetal bovine serum (v/v), 100 ◎M norepinephrine, and 2 mM L-glutamine [2]. Fresh media was added every 2-3 days to maintain a healthy cell population. In a separate set of experiments, GH_3 cells were preincubated with 10 ◎M methylglyoxal (MeG) or 500 U/ml superoxide dismutase (SOD) at 37 °C for 6 hours.

1. Bancroft, F.C. and A.H. Tashjian, Jr., *Control of the production of two protein hormones by rat pituitary cells in culture.* In Vitro, 1970. **6**(3): p. 180-9.
2. Chang, W.T. and S.N. Wu, *Activation of voltage-gated sodium current and inhibition of erg-mediated potassium current caused by telmisartan, an antagonist of angiotensin II type-1 receptor, in HL-1 atrial cardiomyocytes.* Clin Exp Pharmacol Physiol, 2018. **45**(8): p. 797-807.
